# Commitment to the Regulatory T Cell Lineage Requires CARMA1 in the Thymus but Not in the Periphery

**DOI:** 10.1371/journal.pbio.1000051

**Published:** 2009-03-03

**Authors:** Michael J Barnes, Philippe Krebs, Nathaniel Harris, Celine Eidenschenk, Rosana Gonzalez-Quintial, Carrie N Arnold, Karine Crozat, Sosathya Sovath, Eva Marie Moresco, Argyrios N Theofilopoulos, Bruce Beutler, Kasper Hoebe

**Affiliations:** 1 Department of Genetics, The Scripps Research Institute, La Jolla, California, United States of America; 2 Division of Molecular Immunology, Cincinnati Children's Hospital Research Foundation, Cincinnati, Ohio, United States of America; 3 Department of Immunology and Microbial Science, The Scripps Research Institute, La Jolla, California, United States of America; National Jewish Medical and Research Center/Howard Hughes Medical Institute, United States of America

## Abstract

Regulatory T (T_reg_) cells expressing forkhead box P3 (Foxp3) arise during thymic selection among thymocytes with modestly self-reactive T cell receptors. In vitro studies suggest Foxp3 can also be induced among peripheral CD4^+^ T cells in a cytokine dependent manner. T_reg_ cells of thymic or peripheral origin may serve different functions in vivo, but both populations are phenotypically indistinguishable in wild-type mice. Here we show that mice with a *Carma1* point mutation lack thymic CD4^+^Foxp3^+^ T_reg_ cells and demonstrate a cell-intrinsic requirement for CARMA1 in thymic Foxp3 induction. However, peripheral *Carma1-*deficient T_reg_ cells could be generated and expanded in vitro in response to the cytokines transforming growth factor beta (TGFβ) and interleukin-2 (IL-2). In vivo, a small peripheral T_reg_ pool existed that was enriched at mucosal sites and could expand systemically after infection with mouse cytomegalovirus (MCMV). Our data provide genetic evidence for two distinct mechanisms controlling regulatory T cell lineage commitment. Furthermore, we show that peripheral T_reg_ cells are a dynamic population that may expand to limit immunopathology or promote chronic infection.

## Author Summary

In mammals, CD4^+^ T cells are essential for controlling infections, but have the potential to attack host tissues as well, resulting in autoimmune disease. A subset of CD4^+^ T cells, regulatory T cells (T_reg_)—identified by the expression of the forkhead transcription factor *Foxp3*—serve to prevent immunopathology by dampening immune responses. These cells are unique among CD4^+^ T cell subsets, as only the T_reg_ lineage can develop in both the thymus and periphery. Using a genetic approach, we identified a mutation in the gene *Carma1,* a key component of T and B cell signaling, which in mice distinguishes T_reg_ cells derived from the periphery from thymic-derived regulatory T cells. The mutation caused an absence of thymic T_reg_ cells. However, a small population of T_reg_ cells was observed in the spleen, lymph nodes, and colon of *Carma1*-mutant mice that expanded after viral infection, suggesting that peripheral development of T_reg_ cells could still occur. Indeed, *Carma1*-mutant CD4^+^ T cells could be converted into the T_reg_ lineage in vitro. Our results demonstrate an organ-specific requirement for the CARMA1 signaling pathway that developing thymocytes need in order to become T_reg_ cells, but that naïve CD4^+^ T cells can bypass in the periphery. This dichotomy suggests that T_reg_ cells of thymic or peripheral origin may have different specificities or functions in vivo.

## Abstract 3

The organ-specific requirement for CARMA1-dependent signaling in the thymus suggests that regulatory T cells of thymic or peripheral origin may have different roles in vivo.

## Introduction

Two major mechanisms enforce self-tolerance: negative selection in the thymus and dominant tolerance in the periphery. The importance of both mechanisms is underscored by the phenotypes of autoimmune regulator (*Aire*) knockout [1] and *scurfy* mice [2], which have defects in negative selection or dominant tolerance, respectively. Humans with orthologous mutations develop autoimmune polyendocrinopathy-candidiasis-ectodermal dystrophy (APECED) [3,4] or immune dysregulation, polyendocrinopathy, enteropathy, X-linked (IPEX) syndrome [5]. These mutations all result in systemic autoimmunity, though defects in dominant tolerance cause a more severe and fatal disease. The study of dominant tolerance accelerated after cloning of the *scurfy* locus, which identified forkhead box P3 (Foxp3) as an essential molecule [6]. Foxp3 is a transcription factor expressed predominantly in CD4^+^ T cells committed to the regulatory T cell (T_reg_) lineage [7]. Expression of Foxp3 programs T cells with suppressor function, allowing T_reg_ cells to effect dominant tolerance [8].

The majority of T_reg_ cells are derived from the thymus, although an unknown percentage of these cells may develop in the peripheral lymphoid organs. Thymic T_reg_ lineage commitment occurs in CD4 single-positive (SP) thymocytes and requires intermediate affinity binding of the T cell receptor (TCR) [9], co-stimulation through CD80 and CD86 interactions with CD28 [10,11], and the cytokines TGFβ [12] and interleukin (IL)-2 or IL-15 signaling through the shared IL-2Rβ chain [13–16]. Peripheral commitment of naïve CD4^+^ T cells to the T_reg_ lineage, modeled in vitro, requires exogenous TGFβ, in addition to TCR stimulation and concomitant IL-2 production, to induce Foxp3 expression and T_reg_ function [17]. Foxp3 induction can be enhanced in vitro by inhibition of AKT-mediated signaling or transient TCR stimulation [18,19], and may be preferentially driven in vivo by retinoic acid made by macrophages and dendritic cells (DCs) residing in mucosal tissues [20]. The differences in signaling pathways used in the development of thymic versus peripherally induced T_reg_ cells remain largely unexplored.

In this report, we describe the characterization and positional cloning of the *king* mutation. We identified the mutation by screening G3 mice, homozygous for germline mutations induced by *N*-ethyl-*N*-nitrosourea (ENU) [21], to detect defects in T cell effector function. In *king* homozygous mice, no thymic T_reg_ cells were detected, but Foxp3 could be induced among peripheral CD4^+^ T cells in response to cytokines. Thus, the *king* mouse offered a model to explore differences in signaling pathways used for thymic versus peripheral T_reg_ lineage commitment and to study peripheral T_reg_ dynamics, which are normally obscured by the presence of thymic-derived T_reg_ cells. Our studies provide genetic evidence of two pathways, operating in the thymus or periphery, that commit CD4^+^ T cells to the T_reg_ lineage. We also show that viral infection can cause massive expansion of peripheral T_reg_ cells, an event that may reduce immunopathology or contribute to persistent viral infections.

## Results

### Identification of the *king* Mutation

**Figure 1:**
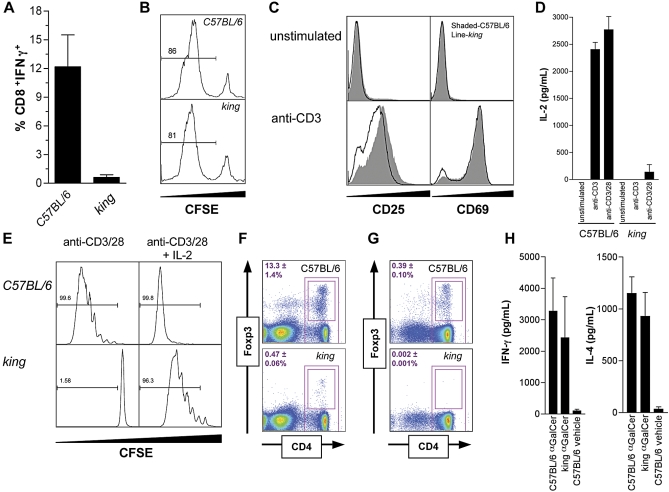
The *king* Mutation Impairs T Cell Activation and T_reg_ Development (A) Activation of CD8^+^ T cells after immunization with γ-irradiated act-mOVA splenocytes. (B) Cross-presentation of ovalbumin epitopes to OT-I T cells by Flt3L-matured bone marrow-derived dendritic cells (BMDCs). (C) Expression of T cell activation markers CD25 and CD69 (intracellular), 24 h after activation of purified CD4^+^ T cells. (D) Production of IL-2 by CD4^+^ T cells 18 h after activation. (E) Proliferation of CD4^+^ T cells, 96 h after activation. (F,G) CD4 and Foxp3 expression in total (F) splenocytes and (G) thymocytes. Standard deviation is indicated. (H) Serum concentrations of IL-4 and IFNγ 90 min after injection with αGalCer. Error bars indicate standard deviation. These experiments were each repeated at least three times with consistent results and representative data are presented.

To identify genes with non-redundant roles in T cell development, priming or effector function, we designed a screen to detect defective cytotoxic CD8^+^ T cell (CTL) responses in mice immunized with ovalbumin (act-mOVA) [22]. Among 2,500 ENU-mutagenized G3 mice screened, we have thus far bred three non-responsive mutations to homozygosity. We termed one of these mutants *king*. While the primary screen used was an in vivo cytotoxicity assay [23], the mutation could be scored using an in vitro assay as well. To do so, we isolated T cells 7 d after immunization, at the peak of the CD8^+^ T cell response, and expanded antigen-specific CD8^+^ T cells in culture with SIINFEKL peptide. *king* CD8^+^ T cells did not undergo secondary expansion or produce interferon (IFN)γ after restimulation with peptide (Figure 1A). We hypothesized that a mutation affecting DC cross-priming of CD8^+^ T cells, T cell activation, or T cell proliferation could cause such a phenotype. To test DC function, we used FMS-like tyrosine kinase 3 (Flt3)-ligand to generate bone marrow-derived lymphoid DCs, a subset of DCs that efficiently cross-primes CD8^+^ T cells [22]. When lymphoid DCs were exposed to ovalbumin expressing apoptotic cells, *king* DCs primed ovalbumin-specific OT-I T cells as efficiently as wild-type DCs (Figure 1B). In addition, *king* DCs showed normal up-regulation of co-stimulatory molecules CD40, CD80, CD86, and major histocompatibility complex (MHC) class I and II after activation by Toll-like receptor ligands [24] or apoptotic cells [22] (unpublished data), suggesting that the mutation did not affect co-stimulation. These results indicated that the *king* mutation did not impair DC-mediated cross-priming of CD8^+^ T cells.

We next investigated T cell activation. *king* CD4^+^ and CD8^+^ T cells normally up-regulated CD69, but not CD25 (IL-2Rα) upon TCR activation (Figure 1C). As CD25 can be further up-regulated in response to IL-2, we measured IL-2 production by *king* CD4^+^ T cells activated by TCR ligation and found a lack thereof (Figure 1D). Since these data implied only a partial defect in T cell activation, we next assessed T cell proliferative capacity. *king* T cells failed to proliferate in response to TCR stimulation, although this could be partially rescued by exogenous IL-2 (Figure 1E). As IL-2 is required to maintain CD4^+^CD25^+^Foxp3^+^ T_reg_ cells in the periphery [14], we assessed the development of these cells in *king* mice. CD4^+^Foxp3^+^ T cells were reduced by an order of magnitude in periphery (Figure 1F), but were absent in the thymus (Figure 1G), indicating that the *king* mutation blocked commitment of developing thymocytes to the T_reg_ lineage. We also investigated the function of another population of self-reactive T cells that develop in the thymus, natural killer T (NKT) cells. 90 min after injection of the NKT cell-specific agonist alpha-galactosylceramide (αGalCer), elevated concentrations of IL-4 and IFNγ were measured in the serum of *king* mice, indicating that the mutation did not impair pan-T cell function (Figure 1H).

Unlike most other mutations that impair T_reg_ development, *king* mice did not exhibit gross signs of autoimmunity. Even in a cohort of *king* mice monitored for over 9 mo, no detectable anti-chromatin auto-antibodies (Figure S1) were found in the serum, nor did the mice develop splenomegaly, lymphoproliferative disease, or signs of chronic inflammation—all aspects of autoimmunity normally controlled by T_reg_ cells.

### *king*, a Mutation in *Carma1*

**Figure 2:**
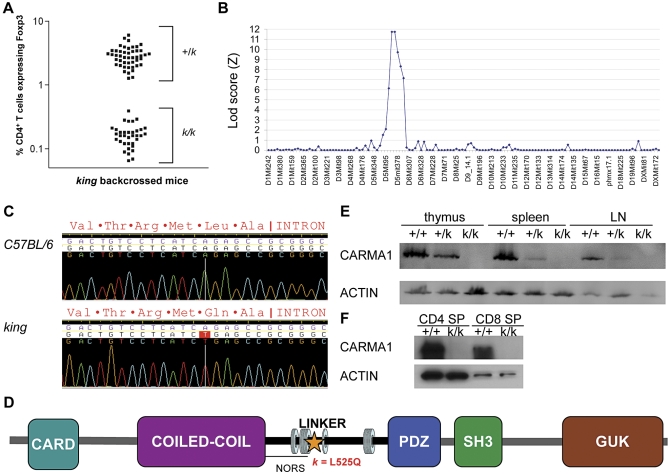
Positional Cloning of the *king* Mutation (A) Percentage of circulating CD4^+^ T cells expressing Foxp3 in *king* homozygous and heterozygous mice. (B) Linkage of the *king* phenotype to Chromosome 5, using 134 informative microsatellite markers. (C) Genomic sequence of *Carma1* in wild-type and *king* mice, with corresponding amino acid sequence. (D) Diagram of the CARMA1 protein (star, *king* mutation; *α*-helices, indicated by rings). Abbreviations: NORS, no regular secondary structure; SH3, Src homology 3; GUK, guanylate kinase. (E) Western blot analysis of CARMA1 expression in thymus, spleen, and lymph node lysates. (F) CARMA1 expression in CD4^+^CD8^−^CD3ɛ^+^ (CD4 SP) and CD4^−^CD8^+^CD3ɛ^+^ (CD8 SP) sorted thymocytes.

To find the causative mutation, we mapped the *king* phenotype by outcrossing the *king* stock (C57BL/6J background) to C3H/HeN mice, backcrossing to the *king* stock, and measuring the percentage of circulating CD4^+^ T cells expressing Foxp3 in the blood of F2 mice (Figure 2A). By analyzing 134 informative microsatellite markers dispersed throughout the genome on 39 meioses, we localized the *king* mutation to the distal region of Chromosome 5 with a peak logarithm of odds (Lod) score of 11.74 (Figure 2B). Further analysis of 268 meioses confined the mutation to a 1.03-Mb critical region, bounded by the markers D5Mit292 and D5Mit101. This region contained only six annotated genes (http://www.informatics.jax.org), and among these was *Card11* (more commonly known as *Carma1* [from CARD-MAGUK1]). We sequenced either genomic DNA or cDNA of all coding basepairs within the critical region and identified a single point mutation in *Carma1* (Figure 2C), which resulted in an L525Q substitution. The mutation occurred in α-helix2, in the NORS (no regular secondary structure) domain, of the CARMA1 linker region (Figure 2D). In naïve T cells, the CARMA1 protein adopts a conformation in which the linker domain associates with the caspase recruitment domain (CARD). Upon T cell activation, protein kinase C (PKC)θ phosphorylation of residues in the linker domain reduces intramolecular affinity for the CARD domain. This liberates both the CARD and coiled-coil domains, allowing CARMA1 oligomerization and recruitment of B cell CLL/lymphoma 10 (BCL10) and mucosa associated lymphoid tissue lymphoma translocation gene 1 (MALT1) to the CARMA1 signaling module [25,26]. Following activation, degradation of BCL10 terminates CARMA1-dependent signaling [27]. CARMA1 has a similar function in B cells, downstream of PKCβ. No CARMA1 expression was detected by western analysis in the thymus, spleen, or lymph nodes of *king* homozygotes (*Carma1^k/k^*) (Figure 2E). Furthermore, CARMA1 was not detectable in CD4 or CD8 SP thymocyte lysates (Figure 2F). The L525Q mutation may have the effect of destabilizing the CARMA1-*king* protein or marking it for degradation in mature T and B cells.

Several other groups have generated targeted knockouts or hypomorphs of *Carma1* [28–31]. Like these other mutant mice, *Carma1^k/k^* mice have reduced basal serum immunoglobulin levels (Figure S2A), fail to mount antigen-specific immunoglobulin (Ig)M and IgG responses after immunization with ovalbumin in Complete Freunds Adjuvant (Figure S2B), and exhibit impaired B cell proliferation (Figure S2C). Lymphocyte development was abnormal in *Carma1^k/k^* mice, as in other *Carma1* mutants, including a deficiency in peritoneal B1 cells and skewed double-negative thymocyte populations (Figure S3A–S3D). Additionally, splenic natural killer (NK) cells, NKT cells, γδ T cells, memory CD4^+^ T cells, and mature B cells were reduced in both percentage and cell number (Figure S3E–S3L and unpublished data). With age, some *Carma1^k/k^* mice developed severe dermatitis, as reported in homozygotes for the *unmodulated* allele of *Carma1* [30]. Collectively, these data suggest that the L525Q *king* mutation abolishes CARMA1 activity, and uncover an essential requirement for CARMA1 in thymic T_reg_ development.

### A Cell-Intrinsic Role for *Carma1* in T_reg_ Lineage Commitment

**Figure 3:**
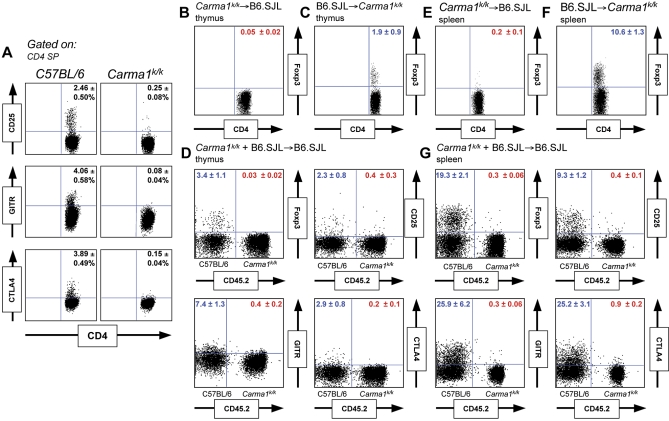
A Cell-Intrinsic Requirement for *Carma1* in T_reg_ Development (A) Expression of T_reg_ markers in CD4 SP thymocytes. (B,C) Foxp3 expression in *Carma1^k/k^* (CD45.2^+^) and C57BL/6.SJL (CD45.2^−^) CD4 SP thymocytes, reconstituted in C57BL/6.SJL (CD45.2^−^) or *Carma1^k/k^* (CD45.2^+^) mice. The percentage of CD4 SP thymocytes expressing Foxp3 is shown. (D) Analysis of T_reg_ markers in wild-type mice reconstituted with mixed *Carma1^k/k^* (CD45.2^+^) and C57BL/6.SJL (CD45.2^−^) bone marrow. The percentage of CD4 SP thymocytes of C57BL/6.SJL or *Carma1^k/k^* origin expressing each marker is shown in blue or red, respectively. (E–G) Using the same mice, the percentage of splenic CD4^+^ T cells expressing Foxp3 in reciprocal (E,F) and mixed (G) bone marrow chimeric mice is shown. B6 denotes C57BL/6.SJL mice. Five reconstituted mice were analyzed for each experiment. Standard deviation is indicated.

In addition to Foxp3, thymocytes committed to the T_reg_ lineage also express CD25, glucocorticoid-induced tumor necrosis factor receptor (GITR), and cytotoxic T-lymphocyte antigen-4 (CTLA-4) [32]. To determine whether *Carma1^k/k^* thymocytes begin differentiation into the T_reg_ lineage but fail to express Foxp3, a distal marker of T_reg_ differentiation, we examined expression of these additional markers (Figure 3A). The lack of expression of these markers suggests CARMA1 acts early in T_reg_ lineage commitment. An absence of thymic T_reg_ cells in *Carma1^k/k^* mice could result from altered selection by thymic epithelial cells, a defect in the TCR and co-stimulatory signaling pathways, or a lack of signaling through the IL-2Rβ chain [13,15,16,33]. To understand the role of *Carma1* in thymic T_reg_ development, we generated reciprocal and mixed bone-marrow chimeric mice. Foxp3 expression was absent in *Carma1^k/k^* thymocytes that developed in a wild-type thymus (Figure 3B), but normal in wild-type thymocytes that developed in a *Carma1^k/k^* thymus (Figure 3C). Therefore, the T_reg_ deficiency in *Carma1^k/k^* mice results not from an altered thymic environment, but rather from an intrinsic defect in hematopoietically derived precursors. When wild-type mice were reconstituted with *Carma1^k/k^* and wild-type bone marrow at a 4:1 ratio, 1:1 chimerism was achieved among lymphocytes. While wild-type thymocytes differentiated into the T_reg_ lineage at normal frequencies, *Carma1^k/k^* thymocytes failed to develop into thymic T_reg_ cells and expressed lower levels of CD25, GITR, and CTLA-4 among CD4 SP thymocytes (Figure 3D). As trans-acting IL-2Rγ chain cytokines produced by wild-type thymocytes did not rescue Foxp3 induction in *Carma1^k/k^* thymocytes, it is likely that impaired signaling downstream of the TCR or CD28 underlies the absence of the thymic T_reg_ cells.

Pathways distinct from those involved in thymic development regulate commitment and homeostasis of peripheral T_reg_ cells [14]. In wild-type mice reconstituted with *Carma1^k/k^* bone marrow, peripheral T_reg_ cells were found at reduced frequencies, similar to those observed in *Carma1^k/k^* mice (Figure 3E). Conversely, in *Carma1^k/k^* mice reconstituted with wild-type bone marrow, peripheral CD4^+^Foxp3^+^ T_reg_ cells occurred at frequencies similar to those observed in wild-type mice (Figure 3F). Therefore, the *Carma1^k/k^* environment can support T_reg_ homeostasis and a cell-intrinsic defect in development causes the T_reg_ deficiency observed in these mice. It has been proposed that IL-2 regulates T_reg_ homeostasis and that T_reg_ cells may function as an IL-2 “sink” [14,34]. However, in wild-type mice reconstituted with mixed wild-type and *Carma1^k/k^* bone marrow, the *Carma1^k/k^* T_reg_ population did not expand in the periphery (Figure 3G). Interestingly, the wild-type T_reg_ population expanded to comprise 20% of the wild-type CD4^+^ T cells, and 10% of the total CD4^+^ T cell pool. This suggests that a cell-extrinsic homeostatic mechanism regulates the size of the T_reg_ compartment in the periphery of naïve wild-type mice.

### TGFβ and IL-2 Drive CARMA1-Independent Foxp3 Induction

**Figure 4:**
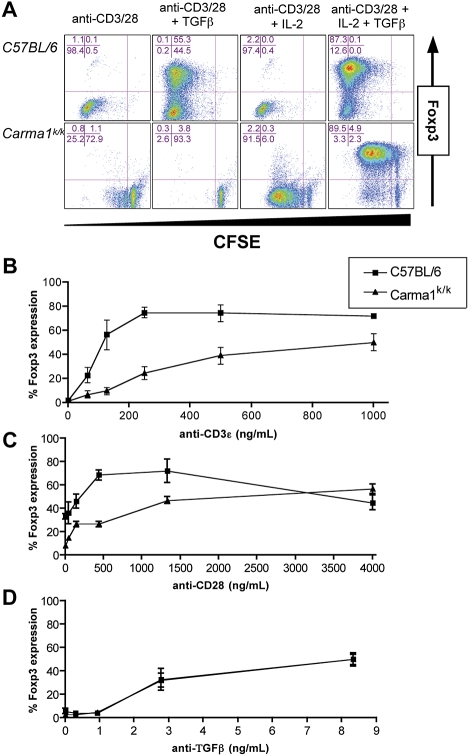
Cytokine Driven Induction of Foxp3 Does Not Require *Carma1* (A) Proliferation and Foxp3 induction in CD4^+^ T cells activated with anti-CD3 (2 μg/ml) and anti-CD28 (2 μg/ml) antibody for 90 h in the presence of IL-2 (100 U/ml) and/or TGFβ (5 ng/ml). (B–D) Dose response analysis of Foxp3 induction in CD4^+^ T cells activated for 90 h with (B) indicated concentrations of anti-CD3 antibody, in the presence of TGFβ (5 ng/ml) and IL-2 (100 U/ml); with (C) indicated concentrations of anti-CD28 antibody, in the presence of anti-CD3 (1 μg/ml), TGFβ (5 ng/ml), and IL-2 (100 U/ml); or with (D) indicated concentrations of TGFβ, in the presence of anti-CD3 (1 μg/ml), anti-CD28 (1 μg/ml), and IL-2 (100 U/ml). These experiments were each repeated at least three times with consistent results and standard deviation is shown.

While *Carma1^k/k^* mice lack thymic T_reg_ cells, they do exhibit a peripheral T_reg_ pool, though it is substantially smaller than that in wild-type mice. Peripheral expansion and conversion of CD4^+^Foxp3^+^ T cells can be modeled in vitro by culturing activated CD4^+^ T cells in the presence of the cytokine TGFβ [17]. When we activated *Carma1^k/k^* CD4^+^ T cells with plate-bound anti-CD3 and anti-CD28 in the presence of exogenous TGFβ, they did not proliferate or express Foxp3. However, the combination of exogenous IL-2 and TGFβ was sufficient to rescue both proliferation and Foxp3 induction (Figure 4A). Importantly, the percentage and number of undivided *Carma1^k/k^* CD4^+^Foxp3^+^ T cells increased, indicating that Foxp3 expression was induced from CD4^+^Foxp3^−^ T cells. Additionally, the induced *Carma1^k/k^* T_reg_ cells did not express stable CARMA1 protein (Figure S4).

To determine whether *Carma1^k/k^* CD4^+^ T cells were more or less prone than wild-type cells to express Foxp3, we preformed a dose-response analysis of Foxp3 induction by titrating anti-CD3, anti-CD28, and TGFβ concentrations. In the absence of CARMA1, the TCR-signaling threshold for Foxp3 induction was increased significantly (Figure 4B). Co-stimulatory signals were also required for efficient Foxp3 induction in wild-type and *Carma1^k/k^* CD4^+^ T cells (Figure 4C). CARMA1 deficiency did not alter the ability of CD4^+^ T cells to respond to limiting concentrations of TGFβ (Figure 4D). These data suggest that Foxp3 induction can occur without CARMA1, and reveal a partial role for CARMA1 in transmitting TCR-mediated signals for peripheral Foxp3 induction.

**Figure 5:**
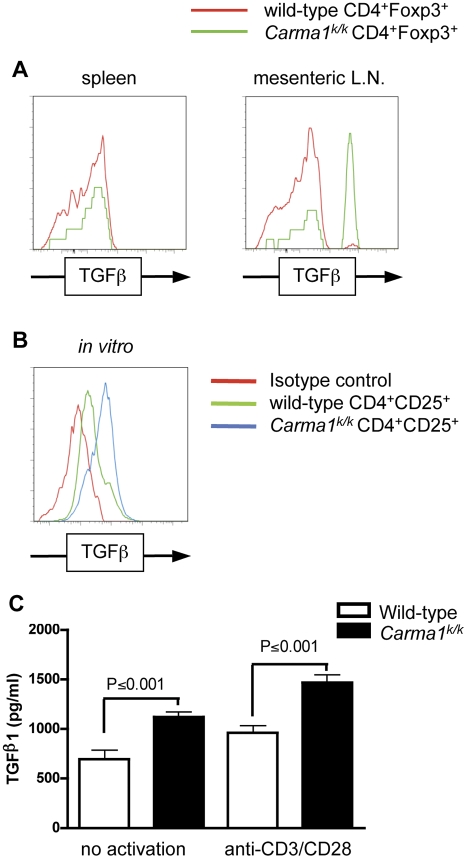
Elevated TGFβ Expression in *Carma1^k/k^* T_reg_ Cells (A,B) Expression of cell associated TGFβ in permeabilized T_reg_ cells (A) in vivo or (B) generated in vitro for 4 d with TGFβ and IL-2. (C) To measure release of soluble TGFβ, equivalent numbers of in vitro generated T_reg_ cells were cultured without exogenous cytokines for 24 h, with or without activation by anti-CD3 and anti-CD28. TGFβ concentration in the supernatant was measured by ELISA.

The absence of detectable thymic T_reg_ cells and presence of a small peripheral T_reg_ population in *Carma1^k/k^* mice suggests that induction of T_reg_ cells can occur in the periphery of naïve mice without activation of the CARMA1 pathway. Also consistent with this conclusion, *Carma1^k/k^* mice had elevated numbers of CD4^+^Foxp3^+^ T cells in the lamina propria and mesenteric lymph nodes (Table 1). The lamina propria of the colon is a site where peripheral conversion of T_reg_ cells may preferentially occur, and these lymphocytes drain to the mesenteric lymph nodes [20,35]. The CD4^+^Foxp3^+^ T_reg_ cells in *Carma1^k/k^* mice showed normal expression of CD25, CTLA-4, and GITR, but interestingly expressed higher amounts of membrane-associated TGFβ in the mesenteric lymph nodes but not in the spleen (Figure 5A). In vitro induced *Carma1^k/k^* T_reg_ cells showed a similar phenotype (Figure 5B) and released more soluble TGFβ than wild-type T_reg_ cells in culture, with or without activation (Figure 5C). Together, these data suggest that peripheral induction of CD4^+^Foxp3^+^ T_reg_ cells occurs in the absence of *Carma1*.

**Table 1:**
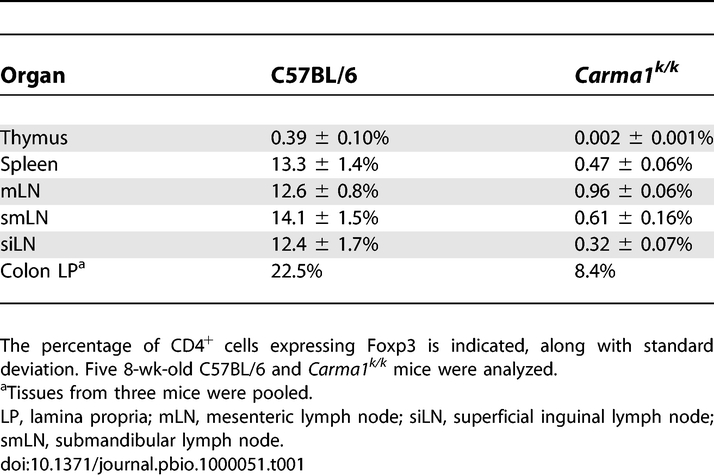
Distribution of T_reg_ Cells in *Carma1^k/k^* Mice

### Characterization of *Carma1*-Independent Foxp3 Induction

**Figure 6:**
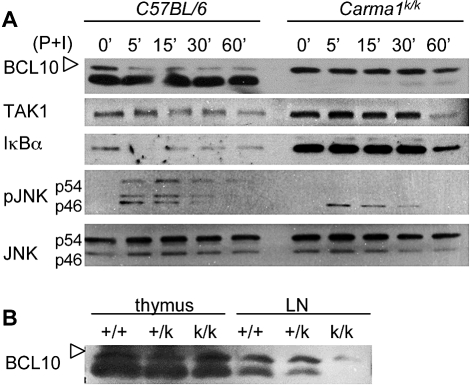
Defective IκB Degradation, JNK Phosphorylation, and BCL10 Stability in *Carma1^k/k^* CD4^+^ T Cells (A) Purified CD4^+^ T cells were activated with PMA and ionomycin for the indicated number of minutes. Expression of indicated proteins and phosphorylated proteins were determined by Western blotting. (B) Expression of BCL10 in thymus and lymph node lysates. Phosphorylated BCL10 is indicated with open arrows. These experiments were each repeated at least three times with consistent results and representative data are presented.

As IL-2 and TGFβ are sufficient to induce Foxp3 in *Carma1^k/k^* CD4^+^ T cells, we explored the possibility that these cytokines activated signaling pathways downstream of CARMA1, or induced Foxp3 expression via an alternative pathway. We first activated wild-type and *Carma1^k/k^* CD4^+^ T cells with the PKC activator phorbol myristate acetate (PMA) and ionomycin, without exogenous cytokines (Figure 6A). Upon activation, BCL10 phosphorylation, NF-κB inhibitor alpha (IκBα) degradation and phosphorylation of both Jun N-terminal kinase (JNK) isoforms occurred in wild-type T cells. In contrast, BCL10 was constitutively phosphorylated, amounts of IκBα were constantly elevated, and the JNKp54 isoform remained unphosphorylated in *Carma1^k/k^* T cells. Interestingly, decreased abundance and constitutive phosphorylation of BCL10 was observed in total *Carma1^k/k^* lymph node T cells, but not thymocytes (Figure 6B). Normally, BCL10 is recruited to CARMA1 after activation. After assembly of the CARMA1 signaling complex, phosphorylation marks BCL10 for ubiquitination and degradation [27]. The lack of CARMA1 protein (Figure 2D) and constitutive degradation of BCL10 in *Carma1^k/k^* T cells indicates that the CARMA1 signaling complex cannot be assembled; this is also reflected by the elevated amounts of TGFβ-activated kinase 1 (TAK1) in resting and activated *Carma1^k/k^* T cells (Figure 6A), likely due to reduced protein turnover.

Other groups have reported that *Carma1*-deficient T cells exhibit a lack of nuclear factor κB (NF-κB) nuclear translocation [28–31] and JNK2 phosphorylation [36] after TCR stimulation. While the CARMA1 signaling complex was inoperative in *Carma1^k/k^* mice, it was possible that exogenous IL-2 and TGFβ retained the ability to activate NF-κB or JNK. However, when we activated wild-type and *Carma1^k/k^* T cells with PMA and ionomycin in the presence of these cytokines, levels of IκBα degradation and JNK phosphorylation remained unchanged up to 60 min after activation (unpublished data). Similar results were obtained when T cells were activated by anti-CD3 and anti-CD28 antibodies.

**Figure 7:**
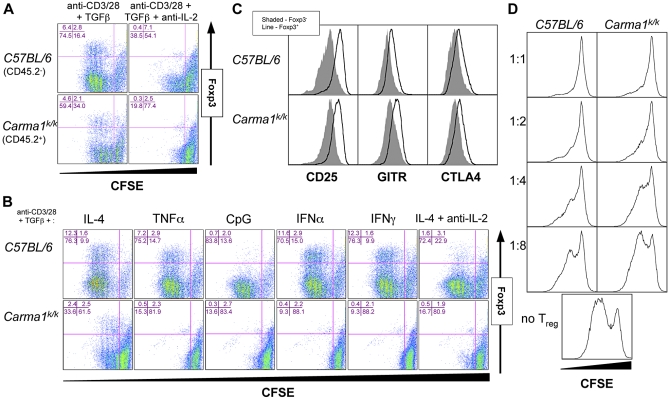
Induced *Carma1^k/k^* T_reg_ Cells Generated with TGFβ and IL-2 Are Functional (A) Co-culture of wild-type and *Carma1^k/k^* CD4^+^ T cells, activated for 70 h, with or without neutralizing IL-2 antibody. (B) Purified CD4^+^ T cells activated for 70 h, in the presence of TGFβ and the indicated molecule. (C) Expression of CD25, GITR, and CTLA-4 in induced T_reg_ cells. (D) Co-culture suppressor assay using wild-type or *Carma1^k/k^* induced T_reg_ cells and CFSE-labeled wild-type CD8^+^ T cells. The ratio of suppressor to responder cells is indicated. CD8^+^ T cell proliferation is shown. These experiments were each repeated at least two times with consistent results and representative data are presented.

We next investigated whether other cytokines could drive Foxp3 expression. To determine if trans-acting cytokines produced by wild-type CD4^+^ T cells could drive Foxp3 induction, we co-cultured activated wild-type and *Carma1^k/k^* CD4^+^ T cells in the presence of TGFβ. The presence of activated wild-type cells allowed proliferation and Foxp3 induction in *Carma1^k/k^* CD4^+^ T cells. Co-culture in the presence of IL-2 neutralizing antibody abolished proliferation of *Carma1^k/k^* CD4^+^ T cells and Foxp3 induction in both wild-type and *Carma1^k/k^* CD4^+^ T cells (Figure 7A). However, other IL-2Rγ chain cytokines can also substitute for exogenous IL-2 in Foxp3 induction [37]. To test whether this could occur in the absence of CARMA1, we activated *Carma1^k/k^* CD4^+^ T cells in the presence of TGFβ and IL-4 (an IL-2Rγ chain cytokine). Exogenous IL-4 induced proliferation and Foxp3 induction, although, as expected, it was less potent than exogenous IL-2 [38]. However, neutralizing IL-2 antibody abrogated this effect (Figure 7B), indicating that other IL-2Rγ chain cytokines can drive IL-2 production and T cell proliferation independently of TCR-mediated CARMA1 activation. It has also been reported that CpG DNA or associated proinflammatory molecules can act, in vitro, directly on T cells to restore TCR-mediated proliferation and induce CD4^+^ T cell polarization in cells from *Pkc*θ*^−/−^* mice [39]. However, neither proliferation nor Foxp3 induction occurred in *Carma1^k/k^* T cells cultured with TGFβ and CpG, TNFα, IFNα, or IFNγ (Figure 7B).

### T_reg_ Cell Suppressor Function Does Not Require CARMA1

It remained unclear whether CARMA1 was required for T_reg_ suppressor function. In addition to Foxp3, in vitro generated T_reg_ cells express CD25, CTLA-4, and GITR at high levels, similar to T_reg_ cells found in vivo. *Carma1^k/k^* T_reg_ cells generated in vitro express normal levels of these markers (Figure 7C). T_reg_ cells have the ability to suppress T cell proliferation by a cell-contact dependent mechanism in vitro [34]. Both wild-type and *Carma1^k/k^* induced T_reg_ cells, generated in vitro with IL-2 and TGFβ, suppressed the proliferation of wild-type CD4^+^ T cells in a co-culture assay (Figure 7D). The difference in observed suppression at 1:4 and 1:8 dilutions likely reflects the proliferation defect in *Carma1^k/k^* T_reg_ cells. These results suggest *Carma1* is neither required for TGFβ-mediated induction of the T_reg_ phenotype, nor for suppressor function.

### C*arma1^k/k^* T_reg_ Cells Expand during Mouse Cytomegalovirus Infection

**Figure 8:**
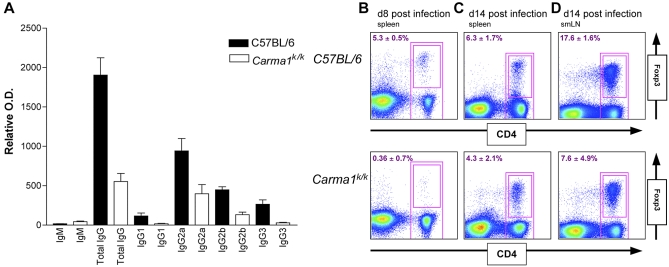
Peripheral Expansion of T_reg_ Cells in *Carma1^k/k^* Mice after MCMV Infection (A) MCMV-specific Ig concentrations measured in serum collected 14 d after infection. (B–D) Foxp3 expression in splenic CD4^+^ T cells 8 (B) or 14 (C) d after infection, and in sub-mandibular lymph node CD4^+^ T cells 14 (D) d after infection. Five mice were analyzed in each group. Standard deviation is indicated.

Peripheral T_reg_ cells comprise a small percentage of *Carma1^k/k^* CD4^+^ T cells in the steady-state. It has been suggested that peripheral T_reg_ cells may expand during conditions of lymphopenia [9], at the site of tumors [40], or in response to pathogens [41]. The absence of thymic-derived T_reg_ cells in *Carma1^k/k^* mice provides a model to study the dynamics of peripheral T_reg_ cells during infection. To do this, we infected *Carma1^k/k^* mice with a pathogen that establishes persistent infection in mice—mouse cytomegalovirus (MCMV). *Carma1^k/k^* mice mounted T-dependent B cell responses that were reduced compared to the wild-type response (Figure 8A), but sufficient to allow survival without any signs of virus-induced immunopathology or detectable virus in the spleen 14 d after infection. Yet, at the peak of the effector CD4^+^ T cell response, 8 d after infection, no T_reg_ expansion was observed in the spleen (Figure 8B). However, splenic CD4^+^Foxp3^+^ T_reg_ cells expanded by an order of magnitude in *Carma1^k/k^* mice 14 d after MCMV infection (Figure 8C). MCMV establishes persistent infection in the salivary glands, and T cells drain from the salivary glands to the submandibular lymph nodes. Here, T_reg_ expansion was also observed in *Carma1^k/k^* mice (Figure 8D). No Foxp3 expression was detected in the thymus 8 or 14 d after infection, suggesting that T_reg_ expansion resulted from either de novo induction of Foxp3 or expansion of the pre-existing peripheral T_reg_ pool. Additionally, MCMV infected *Carma1^k/k^* mice did not develop signs of autoimmune or lymphoproliferative disease when monitored for 80 d after infection.

## Discussion

Accumulating evidence suggests that diverse stimuli can drive proliferation or induction of CD4^+^Foxp3^+^ T_reg_ cells in the periphery [20,40–43]. We have shown that cytokines or MCMV infection can drive Foxp3 induction and peripheral T_reg_ proliferation in the absence of CARMA1, but found different requirements for thymic T_reg_ development. Contrary to expectations that the T_reg_ deficiency observed in mice lacking components of the CARMA1 pathway reflected an inability to produce IL-2 [33], cytokines produced by wild-type thymocytes or thymic epithelial cells did not rescue thymic Foxp3 induction in *Carma1^k/k^* thymocytes in mixed bone marrow chimeric mice. This may reflect the absence of CD4^+^CD25^+^ thymocytes capable of responding to IL-2 and inducing Foxp3 [44] among *Carma1^k/k^* thymocytes. Thus, unlike Foxp3 induction in peripheral CD4^+^ T cells, which can be driven by cytokines without activation of CARMA1, developing thymocytes require activation of a cell-intrinsic, CARMA1-dependent signaling pathway(s) that likely includes the transcription factor NF-κB. CARMA1-mediated NF-κB activation may act as a survival factor in thymic T_reg_ development, preventing apoptosis of thymocytes with certain TCRs or pre-TCRs [28,45] that are destined to become CD4^+^Foxp3^+^ T_reg_ cells.

Given the robust T cell expansion observed in *Carma1^k/k^* mice during MCMV infection, it is perhaps surprising that they do not develop spontaneous autoimmune disease as a consequence of thymic T_reg_ deficiency. Likewise, deletion of other genes in the *Carma1* pathway—*Pkc*θ [46]*, Bcl10* [46]*, Tak1* [47,48], *Ikk*β [49]—impairs T_reg_ development, but does not result in spontaneous autoimmunity (see Figure S5). Mice with a *Ick*-driven CD4-specific deletion of *Tak1* develop colitis with age, although escaped CD4^+^ T cells retaining intact *Tak1* might initiate disease in this model [47]. Additionally, hypomorphic mutations in regulators of TCR-mediated PKCθ activation, linker for T cell activation (*Lat)* [50], and zeta-chain-associated protein kinase 70 (*Zap70*) [51], also block T_reg_ development. In *Carma1^k/k^* mice, T cells, including any with potentially auto-reactive TCRs, are normally quiescent, but can become activated during the “cytokine storm” of infection. The small *Carma1^k/k^* peripheral T_reg_ pool may also contribute to dampening T cell responses directed towards self-antigens or commensal flora and may release more of the regulatory cytokine TGFβ than wild-type T_reg_ cells. Additionally, the impaired B cell function in *Carma1*-deficient mice may prevent amplification of auto-reactive immune responses. We also demonstrated that NKT cell, but not conventional T cell cytokine production occurred normally without functional CARMA1, suggesting that in the absence of thymic-derived T_reg_ cells, *Carma1^k/k^* NKT cells do not drive spontaneous autoimmune disease.

The development of the hematopoietic system and lymphocyte activation defects were similar in *Carma1^k/k^* mice and *Carma1*-knockout mice. Therefore, it was surprising to find reduced protein levels and constitutive phosphorylation of BCL10 in *Carma1^k/k^* CD4^+^ T cells. In CARMA1-deficient JPM50.6 cells, BCL10 expression is normal [52]. The normal expression of BCL10 in *Carma1^k/k^* thymocytes, but not lymph node cells suggests BCL10 degradation may be confined to mature T and B cells. As the *king* mutation occurred in a region of CARMA1 predicted to regulate accessibility of the CARD domain, we propose a model in which BCL10 is constitutively recruited to the CARMA1-*king* protein in mature T and B cells. This interaction is not sufficient to assemble the full CARMA1-BCL10-MALT1 signaling complex or to activate NF-κB, but does allow for BCL10 phosphorylation and the subsequent degradation of BCL10 [27] and the CARMA1-*king* protein. As a result, the CARMA1-BCL10-MALT1 complex cannot be assembled upon TCR stimulation.

The generation of peripheral T_reg_ cells requires TCR signals in addition to TGFβ and IL-2 in vitro. Our data suggest that peripheral Foxp3 induction may not require CARMA1-mediated activation of NF-κB, as indicated by the lack of IκBα degradation or JNK phosphorylation after activation. When IκBα degradation was induced by culturing CD4^+^ T cells with TNFα [29], *Carma1^k/k^* cells still did not proliferate, nor did they up-regulate Foxp3 in the presence of TGFβ. Similar to our observations for *Carma1^k/k^* mice, exogenous IL-2 can also rescue the TCR-mediated proliferation defect in mice deficient for the NF-κB family members *p50* and *c-Rel* [53]. T cells from these mice also cannot produce IL-2, and peripheral CD4^+^CD25^+^ cells are reduced 5-fold. It will be of interest to determine if thymic T_reg_ cells develop in these NF-κB deficient mice and whether peripheral CD4^+^ T cells from these mice induce Foxp3 after exposure to IL-2 and TGFβ.

Recently, two groups reported TGFβ-independent Foxp3 induction in vitro when CD4^+^ T cells were activated in the presence of chemical or genetic inhibitors of the phosphatidylinositol 3-kinase (PI3K)-AKT-mammalian target of rapamycin (mTOR) signaling axis [18,19]. Weak or transient TCR stimulation, without co-stimulation, is also postulated to favor peripheral induction of Foxp3 in vivo [19,43]. However, *Carma1^k/k^* CD4^+^ T cells were less prone than wild-type CD4^+^ T cells to induce Foxp3 when given weak TCR stimulation. Therefore, our data suggest that unlike the AKT signaling axis, blockade of CARMA1-dependent signaling does not favor peripheral Foxp3 induction.

In vivo, we have shown that factors produced during MCMV infection can stimulate peripheral T_reg_ expansion in *Carma1^k/k^* mice. Our in vitro data suggest that this expansion may require IL-2Rγ chain cytokines, such as IL-4, to drive IL-2 production. Expansion of T_reg_ cells may contribute to the transient immunosuppression that can follow viral infection [54], and our data suggest these cells may arise peripherally. Such a response could protect the host against cross-reactive anti-viral T cells that have the potential to precipitate autoimmune disease. An alternative but not mutually exclusive hypothesis holds that MCMV may manipulate the host immune response to expand virus-specific T_reg_ cells. Such a strategy has been well documented for the parasite Leishmania major [42], and may be utilized by other viruses, for example Friend leukaemia virus [41]. If these T_reg_ cells localized to viral reservoirs, they could facilitate persistent viral infections.

The existence of two pathways for Foxp3 induction allows for a total T_reg_ pool with two potential specificities [32]. Thymic T_reg_ cells might primarily express TCRs with intermediate self-affinity [9], as Foxp3 induction would require TCR signals strong enough to activate CARMA1 without causing clonal deletion. Tonic signaling through the TCR along with consumption of cytokines could allow expansion of thymic T_reg_ cells to fill the T_reg_ niche. The generation of peripherally induced T_reg_ cells might be driven by cytokines produced by the innate immune system at mucosal sites or during infection. In *Carma1^−/−^* mice, myeloid cells make normal amounts of IL-2, which may support the peripheral T_reg_ population we observe in *Carma1^k/k^* mice [55]. These induced T_reg_ cells could then prevent T cell responses to commensal flora and dampen potentially dangerous responses to pathogens [41] or innocuous antigens [43].

## Materials and Methods

### Mice, pathogens, and antibodies.

We obtained C57BL/6J and *Jnk2^−/−^* mice from The Jackson Laboratories. We generated *king* mice at TSRI using ENU mutagenesis [21]. They have been made available through the Mutant Mouse Regional Resource Centers (MMRRC:030114-UCD).

The MCMV *smith* strain was isolated from the salivary glands of 3-wk-old infected BALB/c mice. 1 × 10^5^ PFU of MCMV was injected IP per mouse.

The following antibodies were used in this study for flow cytometry: CD4-APC (L3T4), CD25-FITC (PC61.5), CD45.1-FITC (A20), CD45.2-APC (104), CD69-APC (H1.2F3), Foxp3-PE (FJK-16s), GITR-FITC (DTA-1), IFNγ-APC (XMG1.2) (eBioscience); CD4-FITC (L3T4), CTLA-4-FITC (1B8) (Southern Biotech). Intracellular staining was performed for analysis of CTLA-4, Foxp3, and IFNγ. These antibodies were used for western blotting: BCL10 (Santa Cruz); CARD11, IκBα, pJNK, JNK, TAK1 (Cell Signaling). Purified CD3ɛ (145–2C11) and CD28 (37.51) antibodies (eBioscience) were used at indicated concentrations for T cell activation. Carboxyl fluorescent succinimidyl ester (CFSE) labeling was performed by incubating MACS (Miltenyi Biotech) purified CD4^+^ T cells, CD8^+^ OT-I T cells, or splenic B cells in 5 μM CFSE with 0.1% fetal calf serum in PBS for 10 min.

### CD8^+^ T cell functional assays.

To assess the CD8^+^ T cell response, we immunized mice with 1 × 10^7^ γ-irradiated (1,500 rad) act-mOVA splenocytes. 7 d later, 5 × 10^6^ splenocytes were isolated and cultured with 10 nM SIINFEKL peptide in IMDM media supplemented with 10% FCS (Atlanta Bio). 5 d later, cells were restimulated with 100 nM SIINFEKL peptide in the presence of brefeldin A. Production of IFN-γ was assessed by intracellular cytokine staining.

To test cross-priming of CD8^+^ T cells, lymphoid DCs were generated by culturing 1 × 10^7^ bone marrow cells with 200 ng/ml human Flt3-ligand (Peprotech) in supplemented IMDM media for 8 d. 1 × 10^5^ DCs were then co-cultured with 2 × 10^5^ γ-irradiated (1,500 rad) *Kb^−/−^*; act-mOVA splenocytes and 1 × 10^5^ MACS purified CFSE labeled CD8^+^ OT-I T cells. CFSE dilution was assessed 3 d later by flow cytometry.

### NKT cell activation.

NKT cells were activated by injecting mice IV with 2 μg of αGalCer. Serum was collected 90 min later, and cytokine concentration was measured by ELISA (eBioscience).

### In vitro T cell activation.

MACS purified splenic CD4^+^ T cells were used to test T cell activation. To measure up-regulation of activation markers by flow cytometry, cells were activated for 24 h, and then stained for intra-cellular CD25 and CD69. IL-2 production was measured by culturing 2 × 10^5^ cells/ml under activating conditions in supplemented IMDM media. Supernatant was harvested at 18 h and IL-2 was measured by ELISA (eBioscience). T cell proliferation assays entailed activating CFSE-labeled CD4^+^ T cells using 10 μg/ml CD3ɛ and 1 μg/ml CD28 plate-bound antibodies with or without 100 U/ml IL-2. CFSE dilution was measured after 4 d by flow cytometry.

Peripheral T_reg_ cells were generated in 24-well plates by culturing 3 × 10^5^ CFSE-labeled CD4^+^ T cells in plates coated with CD3ɛ and CD28 antibodies with or without IL-2 (R & D Systems) and/or TGFβ (R & D Systems) in supplemented IMDM media. CFSE dilution and intracellular expression of Foxp3 was measured after 4 d of culture. To examine the effect of other cytokines, 2 × 10^5^ CFSE-labeled C57BL/6-CD45.1^+^ and *king*-CD45.2^+^ CD4^+^ T cells were co-cultured in plates coated with 2 μg/ml CD3 and CD28 antibodies in supplemented IMDM media with 5 ng/ml TGFβ and with or without 10 μg/ml neutralizing IL-2 antibody (JES6–1) (eBioscience). Additionally, CFSE dilution and Foxp3 induction was assessed in CFSE-labeled CD4^+^ T cells activated by 2 μg/ml CD3 and CD28 plate-bound antibodies in supplemented IMDM media with 5 ng/ml TGFβ and: 100 nM CpG oligonucleotides (SIGMA), 10 ng/ml IL-4, 10 ng/ml TNFα, 100 U/ml IFNα, or 100 U/ml IFNγ (all cytokines, R & D systems).

For western analysis of T cell signaling, MACS purified CD4^+^ T cells were activated with 50 ng/ml PMA and 500 ng/ml ionomycin (SIGMA) for the indicated time. Cells were lysed in a non-ionic buffer with protease and phosphatase inhibitors for 15 min on ice, then suspended at a 1:1 ratio in Lamelli sample buffer.

### Bone marrow chimeras.

Recipient mice were γ-irradiated (2 × 500 rads) and injected with 1 × 10^8^ donor bone marrow cells. 10 wk later, lymphoid tissues were harvested, homogenized, stained, and analyzed by flow cytometry.

###  In vitro suppressor assays.

The T_reg_ suppressor assay was performed under conditions previously described [34,37]. Briefly, MACS purified CD4^+^ T cells were cultured in plates coated with 2 μg/ml CD3 and CD28 antibodies, in 100 U/ml IL-2 (R & D Systems) and 5 ng/ml TGFβ (R & D Systems) in supplemented IMDM media. After 4 d, CD4^+^ T cells were again MACS purified. Foxp3 induction was assessed by flow cytometry, and in all experiments at least 90% of CD4^+^ T cells expressed Foxp3. Induced T_reg_ cells were harvested and co-cultured at indicated ratios with 5 × 10^4^ MACS purified CFSE-labeled CD8^+^ T cells. Also included were 1 × 10^5^ T cell-depleted, γ-irradiated (3,000 rad) splenocytes as bystander cells and 0.5 μg/ml soluble CD3ɛ antibody. CFSE dilution was assessed by flow cytometry after 3 d of co-culture.

### MCMV-specific Ig measurement.

To measure the concentration of MCMV specific Ig in the serum, 96-well plates were coated with virus, then blocked in 5% milk. Serum samples were diluted 1:200, then serially diluted threefold. Anti-Ig HRP conjugated antibodies were used for detection.

### Accession numbers.

The Entrez GeneID numbers (http://www.ncbi.nlm.nih.gov/sites/entrez?db=gene) for genes and gene products mentioned in the text are: *Aire* (11634), *Akt* (11651), *Bcl10* (12042), *Card11* (108723), *Cd25* (16184), *Cd28* (12487), *Cd3e* (12501), *Cd40* (21939), *Cd69* (12515), *Cd80* (12519), *Cd86* (12524), *c-Rel* (19696), *Ctla-4* (12477), *Flt-3* (14255), *Foxp3* (20371), *Gitr* (21936), *Ifn*α (15960), *Ifn*γ (15978), *Ikb*α (18035), *Ikk*β (16150), *Il-15* (16168), *Il-2* (16183), *Il-2r*β (16185), *Il-2R*γ (16186), *Il-4* (16189), *Jnk2* (26420), *Lat* (16797), *Malt1* (240354), *mTor* (56717), *p50* (18033), *Pi3k* (18708), *Pkc*θ (18761), *Tak1* (26409), *Tgf*β (21803), *Tnf*α (21926), and *Zap70* (22637).

## Supporting Information

Figure S1 Lack of Anti-Chromatin IgG Auto-Antibodies in Aged *king* Mice Serum ELISA measurements from cohorts of homozygous and heterozygous *king* mice, aged for 1.5 or 9 mo (*n* = 5).
(190 KB TIF)

Figure S2 B Cell Function Is Impaired by the *king* Mutation (A) Basal serum immunoglobulin levels in heterozygous (*+*/*k*) and *Carma1^k/k^* (*k*/*k*) mice. A horizontal bar indicates the mean of each column. A paired *t*-test was used to determine statistical significance, *, *p* < 0.05; **, *p* < 0.01.
(B) Serum ELISA measurements of OVA-specific IgG levels 30 d after immunization with CFA-OVA.
(C) Proliferation of splenic B cells 72 h after activation with soluble anti-IgM (10 μg/ml), anti-CD40 (10 μg/ml) + IL-4 (10 ng/ml), or LPS (2 μg/ml).
(676 KB TIF)

Figure S3 Abnormal Lymphocyte Development in *Carma1^k/k^* Mice Analysis of lymphocyte populations in the (A) peritoneal cavity, (B–F) thymus, (G) bone marrow, and (H–L) spleen of 8-wk-old C57BL/6 and *Carma1^k/k^* mice.
(3.39 MB TIF)

Figure S4 CARMA1 Expression in Induced Regulatory T Cells Purified CD4^+^ T cells were activated with anti-CD3 antibodies and cultured with IL-2, with or without TGFβ, for 90 h. Foxp3 induction was assessed by flow cytometry, and CARMA1 protein concentration was measured by western blotting.
(594 KB TIF)

Figure S5 The Role of TCR-Mediated NF-κB Activation in Regulatory T Cell Development Gray ovals highlight genes for which mutant or knockout mice display reduced numbers of CD4^+^CD25^+^ or CD4^+^Foxp3^+^ T_reg_ cells in the steady state. Black ovals represent genes for which knockout mice have normal numbers of CD4^+^Foxp3^+^ T_reg_ cells. The phenotype of mice deficient in genes represented by white circles has not been reported.
(259 KB TIF)

## Abbreviations

αGalCer: alpha-galactosylceramide
BCL10: B cell CLL/lymphoma 10
CARD: caspase recruitment domain
CARMA1: CARD-MAGUK1
CFSE: carboxyl fluorescent succinimidyl ester
CTL: cytotoxic T lymphocyte
CTLA-4: cytotoxic T-lymphocyte antigen 4
DC: dendritic cell
ENU: N-ethyl-N-nitrosourea
FLT3: FMS-like tyrosine kinase 3
Foxp3: forkhead box P3
GITR: glucocorticoid-induced tumor necrosis factor receptor
IκBα: NF-κB inhibitor alpha
IFN: interferon
Ig: immunoglobulin
IL: interleukin
JNK: Jun N-terminal kinase
MALT1: mucosa associated lymphoid tissue lymphoma translocation gene 1
MCMV: mouse cytomegalovirus
NF-κB: nuclear factor κB
NKT cell: natural killer T cell
PKC: protein kinase C
PMA: phorbol myristate acetate
SP: single positive
TAK1: TGFβ-activated kinase 1
TCR: T cell receptor
TGFβ: transforming growth factor beta
TNFα: tumor necrosis factor alpha
T_reg_: regulatory T cell
